# Organically pillared layer framework of [Eu(NH_2_–BDC)(ox)(H_3_O)]

**DOI:** 10.1107/S2056989019014713

**Published:** 2019-11-08

**Authors:** Supaphorn Thammakan, Kitt Panyarat, Apinpus Rujiwatra

**Affiliations:** a Department of Chemistry, Faculty of Science, Chiang Mai University, Chiang Mai, 50200, Thailand; b Materials Science Research Center, Faculty of Science, Chiang Mai University, Chiang Mai 50200, Thailand

**Keywords:** coordination polymer, lanthanide, 2-amino­terephthalic acid, oxalic acid, crystal structure

## Abstract

The non-porous three-dimensional structure of poly[(μ_5_-2-amino­benzene-1,4-di­carboxyl­ato)(μ_6_-oxalato)(oxomium)europium(III)], [Eu(C_8_H_5_NO_4_)(C_2_O_4_)(H_3_O)]_*n*_ or [Eu^III^(NH_2_–BDC)(ox)(H_3_O)]_*n*_ (NH_2_–BDC^2−^ = 2-amino­terephthalate and ox^2−^ = oxalate) is constructed from two-dimensional layers of Eu^III^–carboxyl­ate–oxalate, which are connected by NH_2_–BDC^2−^ pillars. The basic structural unit of the layer is an edge-sharing dimer of *TPRS*-{Eu^III^O_9_}, which is assembled through the ox^2−^ moiety. The intra­layer void is partially occupied by *TPR*-{Eu^III^O_6_} motifs. Weak C—H⋯O and strong, classical intra­molecular N—H⋯O and inter­molecular O—H⋯O hydrogen-bonding inter­actions, as well as weak π–π stacking inter­actions, affix the organic pillars within the framework. The two-dimensional layer can be simplified to a uninodal 4-connected **sql/Shubnikov tetra­gonal plane** net with point symbol {4^4^.6^2^}.

## Abstract 2

An organically pillared Eu^III^–oxalate–carboxyl­ate framework structure with [Eu(NH_2_—BDC)(ox)(H_3_O)] topology is reported. The non-porous three-dimensional structure is constructed from two-dimensional layers of Eu^III^–carboxyl­ate–oxalate, which are pillared by NH_2_—BDC^2−^ pillars. The basic structural unit of the layer is an edge-sharing dimer of *TPRS*-{Eu^III^O_9_}, which is assembled through the ox^2−^ moiety. The intra­layer void is partially occupied by *TPR*-{Eu^III^O_6_} motifs.

## Chemical context   

Lanthanide coordination polymers (LnCPs) have emerged as authentic multifunctional materials finding potential in various applications, *e.g.* magnetism, optics, luminescence and in heterogeneous catalysis (Roy *et al.*, 2014 ▸). In the crystal engineering of LnCPs, the judicious choice of organic ligands is critical. Among the widely employed di­carboxyl­ates, 1,4-benzene­dicarb­oxy­lic acid (H_2_BDC) tends to provide three-dimensional frameworks with permanent porosity. To enhance inter­actions with guest species, additional functional groups can be introduced onto the phenyl ring of BDC^2−^, *e.g*. 2-amino-1,4-benzene­diarboxylic acid (NH_2_-H_2_BDC) in NH_2_-MIL-53(Al) enhanced the carbon dioxide capture capacity (Stavitski *et al.*, 2011 ▸; Flaig *et al.*, 2017 ▸; Wang *et al.*, 2012 ▸). The smallest di­carb­oxy­lic acid, *i.e*. oxalic acid (H_2_ox), on the other hand, may not facilitate the porous framework (Zhang *et al.*, 2016 ▸; Xiahou *et al.*, 2013 ▸) and its presence as a secondary ligand in the fabrication may lead to diversity in the framework structures.
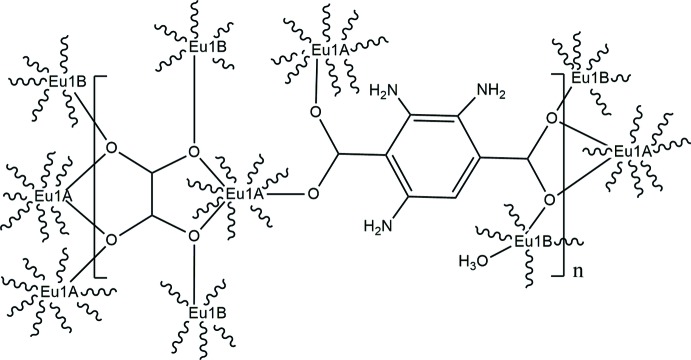


Herein, NH_2_-H_2_BDC and H_2_ox were employed as mixed linkers in the synthesis of a new three-dimensional framework of europium, *i.e.* [Eu(NH_2_-BDC)(ox)(H_3_O)] (**I**). The crystal structure of **I**, which exhibits site disorder at both the Eu^III^ ion and the amino group, is reported. Weak inter­molecular inter­actions and the framework topology are also described.

## Structural commentary   

**Figure 1:**
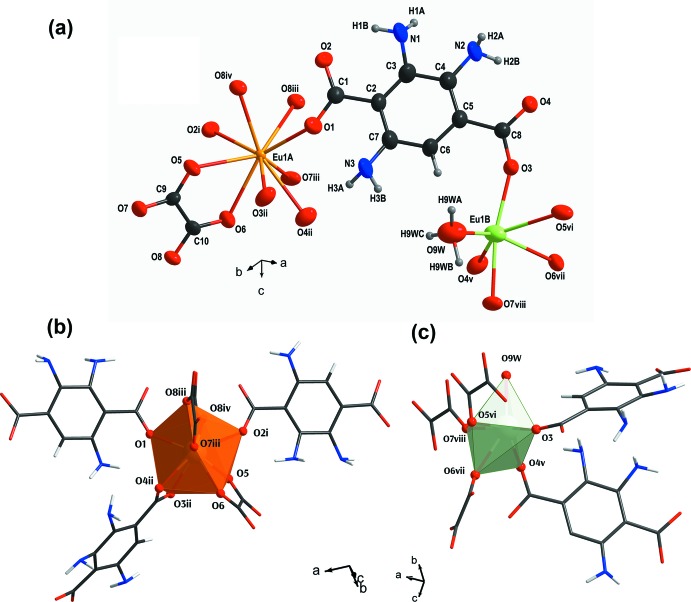
Views of (*a*) an extended asymmetric unit of **I** drawn using 60% probability ellipsoids and the coordination environments about (*b*) Eu1*A* and (*c*) Eu1*B.* [Symmetry codes: (i) −*x*, 1 − *y*, −*z*; (ii) 1 − *x*, 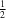 + *y*, 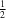 − *z*; (iii) −*x*, −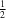 + *y*, 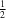 − *z*; (iv) *x*, 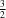 − *y*, −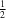 + *z*; (v) *x*, 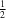 − *y*, 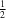 + *z*; (vi) 1 − *x*, −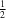 + *y*, 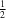 − *z*; (vii) 1 − *x*, 1 − *y*, 1 − *z*; (viii) 1 − *x*, 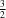 − *y*, 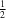 + *z*.]

[Eu(NH_2_–BDC)(ox)(H_3_O)] crystallizes in the monoclinic space group *P*2_1_/*c*. Its asymmetric unit comprises one Eu^III^ ion, which is disordered over two crystallographic sites with an occupying ratio of 0.86 (Eu1*A*): 0.14 (Eu1*B*) and whole mol­ecules of NH_2_–BDC^2−^, ox^2−^ and H_3_O^+^ (Fig. 1 ▸). Eu1*A* is ninefold coordinated to nine O atoms from one chelating NH_2_-–BDC^2−^, two monodentate NH_2_–BDC^2−^, two chelating ox^2−^ and one monodentate ox^2−^ groups, all of which delineate into a distorted tricapped trigonal–prismatic geometry, *i.e. TPRS*-{Eu^III^O_9_}. Eu1*B*, on the other hand, adopts a sixfold coordination of trigonal anti-prismatic geometry, *i.e. TPR*-{Eu^III^O_6_}, which is completed by six O atoms from two monodentate NH_2_–BDC^2−^, three monodentate ox^2−^ and one H_3_O^+^ moieties. Noticeably, the three monodentate ox^2−^ moieties form one trigonal face whereas the two monodentate NH_2_–BDC^2−^ and the ligated H_3_O^+^ moieties outline the other. The Eu^III^—O bond distances ranging between 2.375 (2) and 2.562 (2) Å, are consistent with the values observed for other Eu^III^ coordination frameworks, *e.g*. [(CH_3_)_2_NH_2_]_2_[Eu_6_(μ_3_-OH)_8_(BDC–NH_2_)_6_(H_2_O)_6_] (Yi *et al.*, 2016 ▸) and [Eu_2_(ATPA)_3_(DEF)_2_]_*n*_ where ATPA^2−^ = 2-amino­terepthalate and DEF = di­ethyl­formamide (Kariem *et al.*, 2016 ▸). In addition to the disorder at the Eu^III^ positions, there is an additional disorder at the amino group of NH_2_–BDC^2−^, which distributes over three crystallographic sites with site occupancies of 0.26 (N1), 0.44 (N2) and 0.31 (N3), respectively.

**Figure 2:**
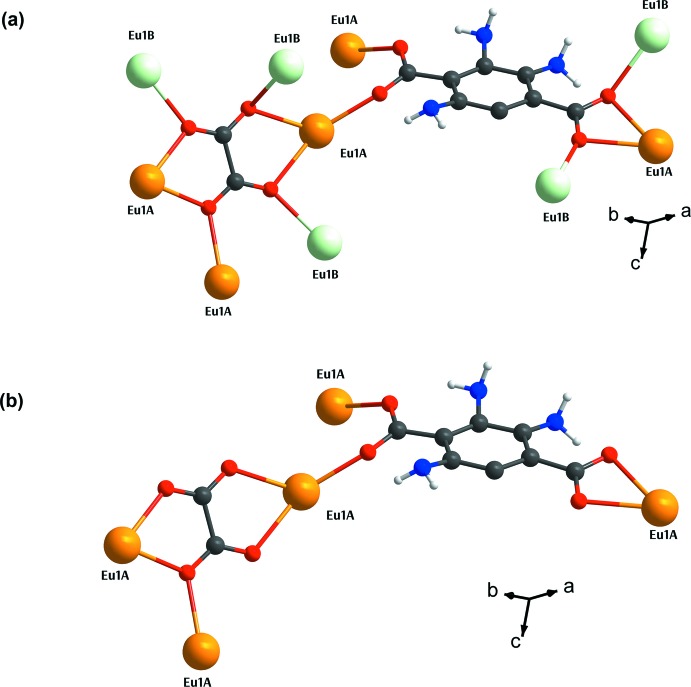
Depictions of coordination modes adopted by NH_2_—BDC^2−^ and ox^2−^; (*a*) with (*b*) without Eu1*B.*

As a result of the disorder of the Eu^III^ ion, the modes of coordinations for both NH_2_–BDC^2−^ and ox^2−^ are diverse. If all of the possible sites of Eu^III^ are concurrently included, the μ_5_-η^1^ *:*η^1^ *:*η^2^ *:*η^2^ mode can be assigned to NH_2_-BDC^2−^ as it connects three Eu1*A* and two Eu1*B* moieties together (Fig. 2 ▸). In a similar fashion, three Eu1*A* and three Eu1*B* moieties may be simultaneously linked by ox^2−^ using the μ_6_-η^2^ *:*η^2^ *:*η^2^ *:*η^2^ mode for coordination. It is worth noting that the μ_5_-η^1^ *:*η^1^ *:*η^2^ *:*η^2^ mode of NH_2_-BDC^2−^ and the μ_6_-η^2^ *:*η^2^ *:*η^2^ *:*η^2^ mode of ox^2−^ are unprecedented. If only the dominating Eu1*A* is regarded, the adopted coordination modes would be μ_3_-η^1^ *:*η^1^ *:*η^1^ *:*η^1^ and μ_3_-η^1^ *:*η^1^:η^1^ *:*η^2^ for NH_2_–BDC^2−^ and ox^2−^, respectively. Likewise, there are only sixteen structures containing ox^2−^ with a μ_3_-η^1^ *:*η^1^:η^1^ *:*η^2^ mode and only two LnCPs comprising NH_2_–BDC^2−^ with a μ_3_-η^1^ *:*η^1^ *:*η^1^ *:*η^1^ mode, *i.e.* [Yb_2_(OH)(atpt)_2.5_(phen)_2_]_*n*_·1.75*n*H_2_O where atpt^2−^ = 2-amino­terephthalate and phen = 1,10-phenanthroline (Liu *et al.*, 2004 ▸), and {[Ho_2_(μ_3_-ATA)_2_(μ_4_-ATA)(H_2_O)_4_]·2DMF·0.5H_2_O}_*n*_ where ATA^2−^ = 2-amino­terephthalate (Almáši *et al.*, 2014 ▸).

## Supra­molecular features   

**Figure 3:**
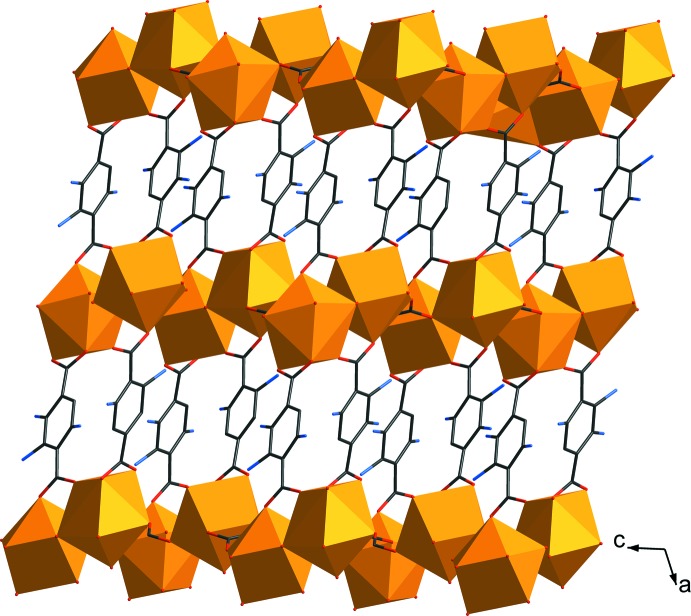
The three-dimensional framework structure of **I**.

**Figure 4:**
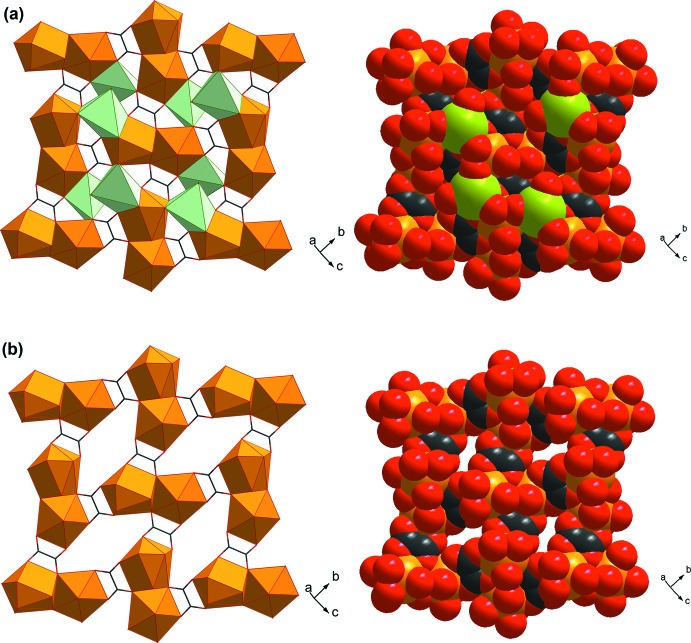
Polyhedral and space-filling representations of the Eu^III^–oxalate–carboxyl­ate layers (*a*) with *TPR*-{Eu^III^O_6_} motifs and (*b*) without *TPR*-{Eu^III^O_6_} motif.

The structure of **I** features a three-dimensional framework, which can be regarded as being built up of two-dimensional layers of Eu^III^-carboxyl­ate-oxalate connected by the NH_2_–BDC^2−^ organic pillars (Fig. 3 ▸). The basic building motif of the layer is the edge-sharing dimer of *TPRS*-{Eu^III^O_9_} (Fig. 4 ▸), which is fused together through two O8 atoms from two ox^2−^ groups and two O1—C1—O2 bridges of two NH_2_-BDC^2−^. Each {Eu_2_^III^O_16_} dimer of Eu1*A* is tied to the other four equivalent dimers through four ox^2−^ linkers in the *bc* plane. The as-described arrangement of these {Eu_2_^III^O_16_} dimers creates voids characterized as the twelve-membered rings, in which the partially occupied *TPR*-{Eu^III^O_6_} motifs of Eu1*B* are situated. Each of the *TPR*-{Eu^III^O_6_} motifs are affixed within the layer through four O atoms from four surrounding ox^2−^ groups and an O4 atom from NH_2_–BDC^2−^. These layers are further connected by the NH_2_–BDC^2−^ organic pillars along the *a-*axis direction providing the non-porous three-dimensional framework. The roles of ox^2−^ and NH_2_–BCD^2−^ in the framework of **I** are, therefore, to create the layer framework and to tether the layers, respectively.

**Figure 5:**
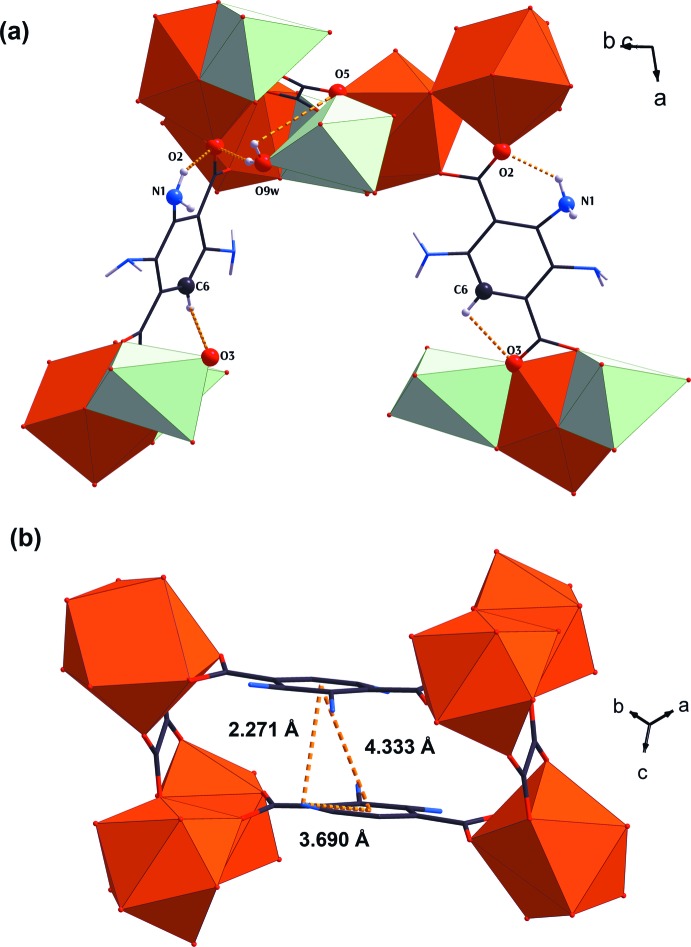
Views of (*a*) the hydrogen-bonding inter­actions and (*b*) the π–π inter­actions.

The NH_2_–BDC^2−^ pillar is apparently organized through intra­molecular hydrogen-bonding inter­actions from both strong N—H⋯O and weak C—H⋯O inter­actions (Table 1 ▸), and through the face-to-face anti­parallel displaced π–π inter­actions (Banerjee *et al.*, 2019 ▸) established between the phenyl rings of two adjacent NH_2_–BDC^2−^ pillars (Fig. 5 ▸). In addition to the intra­molecular hydrogen-bonding inter­actions, two H atoms from the H_3_O^+^ mol­ecule are also involved in providing additional strong O—H⋯O inter­molecular hydrogen-bonding inter­actions.

## Topology   

**Figure 6:**
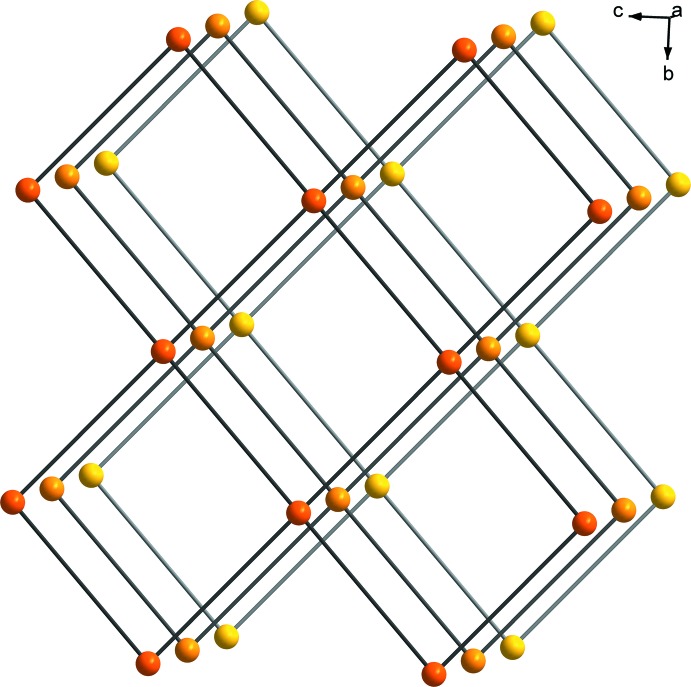
The simplified two- and three-dimensional topologies of **I.**

The topology of the two-dimensional layer of **I** was analysed using *TOPOS* software (Blatov, 2004 ▸). If only the dominating motif, *i.e.* the edge-sharing dimer of Eu1*A*, is taken as a node, which is connected to the other equivalent dimer *via* the ox^2−^ linker, the two-dimensional layer of Eu1 can be simplified to a uninodal 4-connected **sql/Shubnikov tetra­gonal plane** net with a point symbol {4^4^.6^2^} (Blatov *et al.*, 2014 ▸) (Fig. 6 ▸). The inclusion of the partially occupied *TPR*-{Eu^III^O_6_} motifs results in unknown topology. This is also the case for the three-dimensional framework with or without the *TPR*-{Eu^III^O_6_} motifs.

## Photoluminescent property   

A room temperature photoluminescent spectrum of **I** was collected (Jasco FB-8500 spectro­fluoro­meter, λ_excitation_ = 337 nm). It exhibits none of the characteristic *f*–*f* emission of Eu^III^. Even the broad emission characteristic of the ligand-centered π–π emission was not observed. This may be attributed to a proton-induced fluorescence-quenching mechanism facilitated by the presence of H_3_O^+^ in close proximity to the phenyl ring of NH_2_-BDC^2−^ (Tobita & Shizuka, 1980 ▸; Shizuka & Tobita, 1982 ▸). The quenching consequently hinders the sensitization, which is important according to the antenna model (Einkauf *et al.*, 2017 ▸).

## Database survey   

Based on a survey of the Cambridge Structural Database (version 5.40, Nov 2018 with the update of May 2019; Groom *et al.*, 2016 ▸), no LnCP containing both NH_2_-BDC^2−^ and ox^2−^ has previously been reported. However, there are three closely relevant structures which have similar unit-cell parameters, *i.e. catena*-[(μ-tetra­cyano­borate)tetra­aqua­bis­(nitra­to)lanthanum] (Zottnick *et al.*, 2017 ▸), *catena*-[hemikis(pip­erazinedium)(μ-benzene-1,2,4,5-tetra­carboxyl­ato)di­aqua­pras­eo­dymium(III)] (Liang *et al.*, 2017 ▸) and η^5^-inden­yl)di­chloro­tris­(tetra­hydro­furan-*O*)gadolinium tetra­hydro­furan solvate (Fuxing *et al.*, 1992 ▸).

## Synthesis and crystallization   

To synthesize **I**, 2-amino­terephthalic acid (0.2 mmol, 0.0332 g), oxalic acid (0.2 mmol, 0.0180 g) and 1,4-di­aza­bicyclo­[2.2.2]octane (0.4 mmol, 0.0448 g) were dissolved in 8.0 mL of DMF/H_2_O (1 m:7 mL) to prepare solution **A**. Separately, solution **B** was prepared by dissolving Eu_2_O_3_ (0.1 mmol, 0.0180 g) in 1.0 mL of concentrated HNO_3_ aqueous solution, which was then adjusted to pH 7 using a 10 *M* NaOH aqueous solution. Solution **B** was then gradually introduced into solution **A**, and the mixture was then transferred to a 22 mL Teflon-lined stainless-steel autoclave. The reaction was carried out under an autogenous pressure generated at 393 K for 7 days. Yellow crystals of **I** were then recovered by filtration. FT–IR of **I** (KBr; cm^−1^): 3361, 2987, 1617, 1313, 1053, 798.

## Refinement   

Crystal data, data collection and structure refinement details are summarized in Table 2 ▸. The Eu^III^ ion was refined as being disordered over two crystallographic sites resulting in refined occupancies of 0.855 (Eu1*A*) and 0.145 (Eu1*B*). The disorder of the amino group over three crystallographic sites could be clearly seen in the electron-density map and was refined using the SUMP command providing occupancies of 0.259 (N1), 0.440 (N2) and 0.305 (N3). EADP constraints were necessary to make the anisotropic refinements of the disordered N atoms stable. The three H atoms on the ligated H_3_O^+^ (O9*W*) were evident in the electron-density map and therefore assigned as such. The SADI restraint was nonetheless applied on the refinements of the three O—H bonds. H atoms could be positioned from the electron-density maps and were refined as riding with *U*_iso_(H) = 1.2*U*_eq_(C, N) or 1.5*U*_eq_(O). Bond restraints o N—H and O—H were applied in the refunements.

## Supplementary Material

Crystal structure: contains datablock(s) global, I. DOI: 10.1107/S2056989019014713/jj2217sup1.cif

Structure factors: contains datablock(s) I. DOI: 10.1107/S2056989019014713/jj2217Isup2.hkl

CCDC references: 1962371, 1962371

Additional supporting information:  crystallographic information; 3D view; checkCIF report

## Figures and Tables

**Table 1:** Hydrogen-bond geometry (Å, °)

*D*—H⋯*A*	*D*—H	H⋯*A*	*D*⋯*A*	*D*—H⋯*A*
O9*W*—H9*WA*⋯O2^i^	1.11 (5)	1.81 (5)	2.904 (4)	171 (5)
O9*W*—H9*WB*⋯O5^ii^	1.10 (5)	1.87 (5)	2.943 (4)	163 (4)
C6—H6⋯O3	0.93	2.47	2.781 (6)	100
N1—H1*B*⋯O2	0.86	2.03	2.736 (16)	139

Symmetry codes: (i) 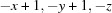; (ii) 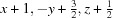.

**Table 2:** Experimental details

Crystal data	
Chemical formula	[Eu(C_8_H_5_NO_4_)(C_2_O_4_)(H_3_O)]
*M*_r_	436.32
Crystal system, space group	Monoclinic, *P*2_1_/*c*
Temperature (K)	293
*a*, *b*, *c* (Å)	11.8348 (3), 11.3208 (3), 10.6531 (3)
β (°)	110.275 (3)
*V* (Å^3^)	1338.86 (7)
*Z*	4
Radiation type	Mo *K*α
μ (mm^−1^)	4.73
Crystal size (mm)	0.2 × 0.05 × 0.05

Data collection	
Diffractometer	Rigaku OD SuperNova, single source at offset/far, HyPix3000
Absorption correction	Multi-scan (*CrysAlis PRO*; Rigaku OD, 2018 ▸)
*T*_min_, *T*_max_	0.753, 0.789
No. of measured, independent and observed [*I* > 2σ(*I*)] reflections	15479, 2882, 2458
*R*_int_	0.050
(sin θ/λ)_max_ (Å^−1^)	0.647

Refinement	
*R*[*F* ^2^ > 2σ(*F* ^2^)], *wR*(*F* ^2^), *S*	0.028, 0.067, 1.09
No. of reflections	2882
No. of parameters	222
No. of restraints	4
H-atom treatment	H atoms treated by a mixture of independent and constrained refinement
Δρ_max_, Δρ_min_ (e Å^−3^)	0.61, −0.54

Computer programs: *CrysAlis PRO* (Rigaku OD, 2018 ▸), *SHELXT* (Sheldrick, 2015*b* ▸), *SHELXL* (Sheldrick, 2015*a* ▸) and *OLEX2* (Dolomanov *et al.*, 2009 ▸).

## supplementary crystallographic information

### Crystal data

**Table:**

[Eu(C_8_H_5_NO_4_)(C_2_O_4_)(H_3_O)]	*F*(000) = 832
*M**_r_* = 436.32	*D*_x_ = 2.165 Mg m^−^^3^
Monoclinic, *P*2_1_/*c*	Mo *K*α radiation, λ = 0.71073 Å
*a* = 11.8348 (3) Å	Cell parameters from 9488 reflections
*b* = 11.3208 (3) Å	θ = 1.8–27.3°
*c* = 10.6531 (3) Å	µ = 4.73 mm^−^^1^
β = 110.275 (3)°	*T* = 293 K
*V* = 1338.86 (7) Å^3^	Block, clear light yellow
*Z* = 4	0.2 × 0.05 × 0.05 mm

### Data collection

**Table:**

Rigaku OD SuperNova, single source at offset/far, HyPix3000 diffractometer	2458 reflections with *I* > 2σ(*I*)
Radiation source: micro-focus sealed X-ray tube	*R*_int_ = 0.050
ω scans	θ_max_ = 27.4°, θ_min_ = 1.8°
Absorption correction: multi-scan (CrysAlis PRO; Rigaku OD, 2018)	*h* = −14→15
*T*_min_ = 0.753, *T*_max_ = 0.789	*k* = −11→14
15479 measured reflections	*l* = −13→13
2882 independent reflections	

### Refinement

**Table:**

Refinement on *F*^2^	4 restraints
Least-squares matrix: full	Hydrogen site location: mixed
*R*[*F*^2^ > 2σ(*F*^2^)] = 0.028	H atoms treated by a mixture of independent and constrained refinement
*wR*(*F*^2^) = 0.067	*w* = 1/[σ^2^(*F*_o_^2^) + (0.0305*P*)^2^ + 0.0276*P*] where *P* = (*F*_o_^2^ + 2*F*_c_^2^)/3
*S* = 1.09	(Δ/σ)_max_ = 0.001
2882 reflections	Δρ_max_ = 0.61 e Å^−^^3^
222 parameters	Δρ_min_ = −0.54 e Å^−^^3^

### Special details

**Table:**

Geometry. All esds (except the esd in the dihedral angle between two l.s. planes) are estimated using the full covariance matrix. The cell esds are taken into account individually in the estimation of esds in distances, angles and torsion angles; correlations between esds in cell parameters are only used when they are defined by crystal symmetry. An approximate (isotropic) treatment of cell esds is used for estimating esds involving l.s. planes.

### Fractional atomic coordinates and isotropic or equivalent isotropic displacement parameters (Å^2^)

**Table:**

	*x*	*y*	*z*	*U*_iso_*/*U*_eq_	Occ. (<1)
Eu1A	0.07429 (2)	0.61284 (2)	0.15674 (2)	0.01311 (9)	0.8558 (8)
O6	0.0486 (2)	0.7311 (2)	0.3456 (2)	0.0298 (6)	
O8	−0.0221 (2)	0.89620 (19)	0.4069 (3)	0.0248 (6)	
O5	−0.0175 (2)	0.8135 (2)	0.0928 (2)	0.0314 (6)	
O2	0.1449 (2)	0.4078 (2)	−0.1090 (3)	0.0315 (7)	
O7	−0.0772 (2)	0.9814 (2)	0.1603 (2)	0.0298 (6)	
O4	0.7236 (2)	0.1331 (2)	0.1432 (3)	0.0356 (7)	
O1	0.2130 (2)	0.5301 (3)	0.0649 (3)	0.0385 (7)	
O3	0.7668 (2)	0.2640 (2)	0.3050 (3)	0.0366 (7)	
C10	0.0008 (3)	0.8307 (3)	0.3236 (4)	0.0212 (8)	
C1	0.2274 (3)	0.4463 (4)	−0.0068 (4)	0.0278 (9)	
C9	−0.0344 (3)	0.8795 (3)	0.1784 (4)	0.0229 (9)	
C8	0.6941 (3)	0.2224 (4)	0.1960 (4)	0.0290 (9)	
C2	0.3499 (3)	0.3911 (3)	0.0360 (4)	0.0319 (10)	
C5	0.5750 (3)	0.2802 (4)	0.1344 (4)	0.0353 (10)	
C6	0.5557 (4)	0.3900 (4)	0.1808 (5)	0.0502 (14)	
H6	0.619345	0.426877	0.246414	0.060*	
C3	0.3677 (4)	0.2839 (4)	−0.0150 (5)	0.0513 (13)	
C7	0.4454 (4)	0.4470 (4)	0.1332 (5)	0.0480 (12)	
C4	0.4805 (4)	0.2271 (4)	0.0321 (5)	0.0528 (13)	
N2	0.4834 (8)	0.1125 (8)	−0.0128 (12)	0.066 (3)	0.439 (6)
H2A	0.512716	0.113790	−0.078500	0.080*	0.439 (6)
H2B	0.531521	0.063870	0.047841	0.080*	0.439 (6)
N3	0.4511 (10)	0.5604 (13)	0.1717 (14)	0.066 (3)	0.312 (6)
H3A	0.407927	0.583997	0.230300	0.080*	0.312 (6)
H3B	0.527520	0.590092	0.221251	0.080*	0.312 (6)
N1	0.2993 (12)	0.2381 (15)	−0.1365 (15)	0.066 (3)	0.261 (6)
H1A	0.286630	0.164575	−0.125872	0.080*	0.261 (6)
H1B	0.231669	0.274954	−0.165662	0.080*	0.261 (6)
Eu1B	0.82330 (16)	0.39654 (13)	0.47691 (17)	0.0427 (7)	0.1442 (8)
O9W	0.8080 (3)	0.5707 (3)	0.3580 (3)	0.0593 (9)	
H9WA	0.816 (5)	0.581 (4)	0.258 (4)	0.089*	
H9WB	0.881 (4)	0.620 (4)	0.433 (5)	0.089*	
H9WC	0.725 (4)	0.578 (6)	0.383 (7)	0.15 (3)*	

### Atomic displacement parameters (Å^2^)

**Table:**

	*U*^11^	*U*^22^	*U*^33^	*U*^12^	*U*^13^	*U*^23^
Eu1A	0.01489 (13)	0.00998 (14)	0.01356 (13)	−0.00037 (7)	0.00379 (9)	−0.00035 (8)
O6	0.0446 (16)	0.0207 (15)	0.0244 (14)	0.0096 (12)	0.0121 (12)	0.0042 (12)
O8	0.0355 (15)	0.0197 (15)	0.0206 (15)	0.0018 (11)	0.0114 (13)	−0.0022 (11)
O5	0.0494 (17)	0.0224 (16)	0.0230 (14)	0.0099 (13)	0.0133 (13)	0.0000 (12)
O2	0.0215 (14)	0.0420 (19)	0.0291 (16)	−0.0002 (12)	0.0064 (12)	−0.0073 (13)
O7	0.0425 (16)	0.0182 (15)	0.0244 (14)	0.0085 (12)	0.0060 (12)	0.0020 (12)
O4	0.0320 (16)	0.0368 (18)	0.0329 (17)	0.0101 (12)	0.0045 (13)	−0.0070 (13)
O1	0.0243 (14)	0.048 (2)	0.0420 (17)	0.0037 (13)	0.0095 (13)	−0.0161 (15)
O3	0.0306 (15)	0.0333 (18)	0.0357 (17)	0.0088 (13)	−0.0014 (13)	−0.0089 (14)
C10	0.0242 (19)	0.014 (2)	0.026 (2)	−0.0034 (16)	0.0088 (16)	−0.0023 (17)
C1	0.025 (2)	0.030 (2)	0.029 (2)	−0.0008 (18)	0.0107 (18)	−0.0008 (19)
C9	0.026 (2)	0.021 (2)	0.017 (2)	0.0012 (16)	0.0008 (17)	0.0012 (16)
C8	0.028 (2)	0.027 (2)	0.028 (2)	0.0023 (18)	0.0054 (18)	−0.0028 (19)
C2	0.025 (2)	0.033 (3)	0.035 (2)	0.0063 (17)	0.0074 (19)	−0.0015 (19)
C5	0.030 (2)	0.035 (3)	0.036 (2)	0.0084 (18)	0.005 (2)	−0.004 (2)
C6	0.031 (3)	0.044 (3)	0.056 (3)	0.013 (2)	−0.010 (2)	−0.020 (2)
C3	0.031 (2)	0.048 (3)	0.058 (3)	0.009 (2)	−0.006 (2)	−0.017 (3)
C7	0.035 (2)	0.046 (3)	0.052 (3)	0.015 (2)	0.001 (2)	−0.016 (3)
C4	0.042 (3)	0.053 (3)	0.049 (3)	0.018 (2)	−0.003 (2)	−0.019 (3)
N2	0.033 (4)	0.064 (5)	0.081 (6)	0.026 (3)	−0.007 (4)	−0.040 (4)
N3	0.033 (4)	0.064 (5)	0.081 (6)	0.026 (3)	−0.007 (4)	−0.040 (4)
N1	0.033 (4)	0.064 (5)	0.081 (6)	0.026 (3)	−0.007 (4)	−0.040 (4)
Eu1B	0.0590 (12)	0.0319 (11)	0.0319 (10)	−0.0013 (7)	0.0090 (8)	−0.0028 (7)
O9W	0.076 (3)	0.056 (2)	0.044 (2)	−0.014 (2)	0.0174 (19)	0.0008 (19)

### Geometric parameters (Å, º)

**Table:**

Eu1A—Eu1A^i^	4.0868 (4)	O4—C8	1.264 (4)
Eu1A—O6	2.521 (2)	O4—Eu1B^vii^	2.468 (3)
Eu1A—O8^ii^	2.562 (2)	O1—C1	1.266 (4)
Eu1A—O8^iii^	2.508 (3)	O3—C8	1.272 (4)
Eu1A—O5	2.508 (2)	O3—Eu1B	2.281 (3)
Eu1A—O2^i^	2.476 (2)	C10—C9	1.558 (5)
Eu1A—O7^ii^	2.444 (3)	C1—C2	1.497 (5)
Eu1A—O4^iv^	2.604 (3)	C8—C5	1.484 (5)
Eu1A—O1	2.375 (2)	C2—C3	1.375 (5)
Eu1A—O3^iv^	2.470 (3)	C2—C7	1.393 (6)
O6—C10	1.247 (4)	C5—C6	1.386 (6)
O6—Eu1B^v^	2.445 (3)	C5—C4	1.398 (6)
O8—C10	1.256 (4)	C6—C7	1.386 (6)
O5—C9	1.247 (4)	C3—C4	1.408 (6)
O5—Eu1B^iv^	2.811 (3)	C3—N1	1.368 (14)
O2—C1	1.261 (4)	C7—N3	1.343 (13)
O7—C9	1.247 (4)	C4—N2	1.387 (8)
O7—Eu1B^vi^	2.349 (3)	Eu1B—O9W	2.317 (4)
			
O6—Eu1A—Eu1A^i^	147.91 (6)	Eu1B^vi^—O7—Eu1A^ix^	99.74 (10)
O6—Eu1A—O8^ii^	129.40 (8)	C8—O4—Eu1A^x^	91.4 (2)
O6—Eu1A—O4^iv^	68.33 (9)	C8—O4—Eu1B^vii^	134.7 (3)
O8^iii^—Eu1A—Eu1A^i^	36.75 (5)	Eu1B^vii^—O4—Eu1A^x^	92.50 (9)
O8^ii^—Eu1A—Eu1A^i^	35.85 (6)	C1—O1—Eu1A	143.2 (2)
O8^iii^—Eu1A—O6	137.05 (8)	C8—O3—Eu1A^x^	97.5 (2)
O8^iii^—Eu1A—O8^ii^	72.60 (9)	C8—O3—Eu1B	153.3 (3)
O8^ii^—Eu1A—O4^iv^	111.70 (8)	Eu1B—O3—Eu1A^x^	109.19 (11)
O8^iii^—Eu1A—O4^iv^	145.39 (9)	O6—C10—O8	126.6 (3)
O5—Eu1A—Eu1A^i^	108.73 (6)	O6—C10—C9	117.1 (3)
O5—Eu1A—O6	64.84 (8)	O8—C10—C9	116.3 (3)
O5—Eu1A—O8^iii^	75.76 (8)	O2—C1—O1	123.6 (3)
O5—Eu1A—O8^ii^	138.85 (8)	O2—C1—C2	119.8 (4)
O5—Eu1A—O4^iv^	109.32 (8)	O1—C1—C2	116.6 (3)
O2^i^—Eu1A—Eu1A^i^	69.48 (6)	O5—C9—C10	117.2 (3)
O2^i^—Eu1A—O6	78.84 (8)	O7—C9—O5	126.9 (4)
O2^i^—Eu1A—O8^iii^	73.70 (9)	O7—C9—C10	115.9 (3)
O2^i^—Eu1A—O8^ii^	73.49 (8)	O4—C8—Eu1A^x^	63.02 (19)
O2^i^—Eu1A—O5	72.87 (8)	O4—C8—O3	119.9 (3)
O2^i^—Eu1A—O4^iv^	140.91 (9)	O4—C8—C5	121.5 (3)
O7^ii^—Eu1A—Eu1A^i^	98.87 (6)	O3—C8—Eu1A^x^	56.94 (18)
O7^ii^—Eu1A—O6	70.07 (9)	O3—C8—C5	118.6 (4)
O7^ii^—Eu1A—O8^ii^	64.12 (8)	C5—C8—Eu1A^x^	174.2 (3)
O7^ii^—Eu1A—O8^iii^	134.20 (8)	C3—C2—C1	120.9 (4)
O7^ii^—Eu1A—O5	130.79 (9)	C3—C2—C7	119.9 (4)
O7^ii^—Eu1A—O2^i^	80.22 (9)	C7—C2—C1	119.1 (4)
O7^ii^—Eu1A—O4^iv^	69.26 (8)	C6—C5—C8	119.1 (4)
O7^ii^—Eu1A—O3^iv^	119.41 (9)	C6—C5—C4	118.5 (4)
O4^iv^—Eu1A—Eu1A^i^	137.47 (6)	C4—C5—C8	122.4 (4)
O1—Eu1A—Eu1A^i^	65.16 (6)	C5—C6—C7	122.5 (4)
O1—Eu1A—O6	146.09 (8)	C2—C3—C4	121.2 (4)
O1—Eu1A—O8^ii^	69.59 (9)	N1—C3—C2	125.9 (7)
O1—Eu1A—O8^iii^	70.84 (8)	N1—C3—C4	109.9 (7)
O1—Eu1A—O5	122.76 (9)	C6—C7—C2	118.6 (4)
O1—Eu1A—O2^i^	134.63 (9)	N3—C7—C2	127.1 (6)
O1—Eu1A—O7^ii^	105.55 (9)	N3—C7—C6	113.1 (6)
O1—Eu1A—O4^iv^	78.60 (9)	C5—C4—C3	119.0 (4)
O1—Eu1A—O3^iv^	75.28 (9)	N2—C4—C5	124.2 (5)
O3^iv^—Eu1A—Eu1A^i^	130.96 (7)	N2—C4—C3	116.0 (5)
O3^iv^—Eu1A—O6	78.21 (9)	O6^v^—Eu1B—O5^x^	70.24 (9)
O3^iv^—Eu1A—O8^iii^	104.11 (8)	O6^v^—Eu1B—O4^xi^	71.76 (10)
O3^iv^—Eu1A—O8^ii^	143.79 (9)	O7^xii^—Eu1B—O6^v^	72.95 (10)
O3^iv^—Eu1A—O5	69.54 (8)	O7^xii^—Eu1B—O5^x^	101.31 (10)
O3^iv^—Eu1A—O2^i^	141.55 (9)	O7^xii^—Eu1B—O4^xi^	73.14 (10)
O3^iv^—Eu1A—O4^iv^	51.17 (8)	O4^xi^—Eu1B—O5^x^	141.46 (10)
C10—O6—Eu1A	120.2 (2)	O3—Eu1B—O6^v^	99.37 (11)
C10—O6—Eu1B^v^	143.1 (2)	O3—Eu1B—O5^x^	66.84 (9)
C10—O8—Eu1A^viii^	126.6 (2)	O3—Eu1B—O7^xii^	167.83 (13)
C10—O8—Eu1A^ix^	118.0 (2)	O3—Eu1B—O4^xi^	114.01 (12)
C9—O5—Eu1A	120.6 (2)	O3—Eu1B—O9W	100.09 (12)
C9—O5—Eu1B^iv^	110.2 (2)	O9W—Eu1B—O6^v^	146.74 (13)
C1—O2—Eu1A^i^	130.7 (2)	O9W—Eu1B—O5^x^	93.23 (12)
C9—O7—Eu1A^ix^	123.2 (2)	O9W—Eu1B—O7^xii^	82.84 (12)
C9—O7—Eu1B^vi^	137.1 (2)	O9W—Eu1B—O4^xi^	122.82 (13)

Symmetry codes: (i) −*x*, −*y*+1, −*z*; (ii) −*x*, *y*−1/2, −*z*+1/2; (iii) *x*, −*y*+3/2, *z*−1/2; (iv) −*x*+1, *y*+1/2, −*z*+1/2; (v) −*x*+1, −*y*+1, −*z*+1; (vi) *x*−1, −*y*+3/2, *z*−1/2; (vii) *x*, −*y*+1/2, *z*−1/2; (viii) *x*, −*y*+3/2, *z*+1/2; (ix) −*x*, *y*+1/2, −*z*+1/2; (x) −*x*+1, *y*−1/2, −*z*+1/2; (xi) *x*, −*y*+1/2, *z*+1/2; (xii) *x*+1, −*y*+3/2, *z*+1/2.

### Hydrogen-bond geometry (Å, º)

**Table:**

*D*—H···*A*	*D*—H	H···*A*	*D*···*A*	*D*—H···*A*
O9*W*—H9*WA*···O2^xiii^	1.11 (5)	1.81 (5)	2.904 (4)	171 (5)
O9*W*—H9*WB*···O5^xii^	1.10 (5)	1.87 (5)	2.943 (4)	163 (4)
C6—H6···O3	0.93	2.47	2.781 (6)	100
N1—H1*B*···O2	0.86	2.03	2.736 (16)	139

Symmetry codes: (xii) *x*+1, −*y*+3/2, *z*+1/2; (xiii) −*x*+1, −*y*+1, −*z*.
